# Supplementary material for: A fault-intrusion-tolerant system and deadline-aware algorithm for scheduling scientific workflow in the cloud
Source: PeerJ Comput Sci. 2021 Nov 2;7:e747. doi: 10.7717/peerj-cs.747 (PMC8576554; doi:10.7717/peerj-cs.747)
Supplement: Supplemental Information 6 [file peerj-cs-07-747-s006.zip › Myproject1.1/docs/org/workflowsim/clustering/balancing/metrics/class-use/BalancingMetric.html]

Uses of Interface org.workflowsim.clustering.balancing.metrics.BalancingMetric


JavaScript is disabled on your browser.


- Overview
- Package
- Class
- Use
- Tree
- Deprecated
- Index
- Help

- Prev
- Next

- Frames
- No Frames

- All Classes

## Uses of Interface org.workflowsim.clustering.balancing.metrics.BalancingMetric

- Packages that use BalancingMetric

  | Package | Description |
  | --- | --- |
  |  |  |
  | --- | --- |
  | org.workflowsim.clustering.balancing.metrics |  |
- - ### Uses of BalancingMetric in org.workflowsim.clustering.balancing.metrics

    Classes in org.workflowsim.clustering.balancing.metrics that implement BalancingMetric

    | Modifier and Type | Class and Description |
    | --- | --- |
    |  |  |
    | --- | --- |
    | `class` | `DistanceVariance` A DistanceVariance |
    | `class` | `HorizontalRuntimeVariance` HorizontalRuntimeVariance is the standard deviation of the runtime |
    | `class` | `ImpactFactorVariance` ImpactFactorVariance is the standard deviation of their impact factors |
    | `class` | `PipelineRuntimeVariance` The PipelineRuntimeVariance is the standard deviation of the runtime of the pipelines |

- Overview
- Package
- Class
- Use
- Tree
- Deprecated
- Index
- Help

- Prev
- Next

- Frames
- No Frames

- All Classes
